# Outcomes of apple oral immunotherapy in pollen food allergy syndrome

**DOI:** 10.1016/j.jacig.2024.100271

**Published:** 2024-05-01

**Authors:** Desie Dijkema, Mirte C. Ruitenbeek, Kirsten Weerstand-Noor, Hanneke N.G. Oude Elberink, Annick A.J.M. van de Ven

**Affiliations:** a Department of Dietetics, University Medical Centre Groningen, Groningen, The Netherlands; b Department of Internal Medicine, Division of Allergology, University Medical Centre Groningen, Groningen, The Netherlands; c Department of Rheumatology and Clinical Immunology, University Medical Centre Groningen, Groningen, The Netherlands

**Keywords:** Oral immunotherapy, Bet v 1, apple, pollen food allergy syndrome, oral allergy

## Abstract

Oral immunotherapy with apple induces tolerance for an entire apple (128 g) in patients with pollen food allergy syndrome who previously tolerated a median amount of 4 g of apple.

Up to 70% of patients with birch pollen allergic may develop pollen food allergy syndrome (PFAS).^1^ Patients may react to tree nuts and fruit of the Rosaceae family, including apple, as these contain food allergens homologous to *Betula verrucosa* 1 (Bet v 1) of birch pollen. Allergic rhinoconjunctivitis symptoms can be ameliorated by using subcutaneous or sublingual allergen immunotherapy, but its effects on PFAS are limited and inconsistent.^2^^,^^3^ Oral immunotherapy (OIT) for primary food allergies has been successful in experimental settings, but little data regarding PFAS are available. Experience with apple OIT is scarce but has shown promising results; 44% to 63% of patients who undertook apple OIT were able to tolerate a whole apple (128 g) after an average of 20 weeks^4^ to 8 months.^5^

Apple OIT has been successfully used at our department, and on the basis of our clinical experiences, a novel protocol was implemented. Here we have described the outcomes in the participants for the first 6 months. The need for informed consent was waived by the local ethical committee. Patients with a confirmed allergy to birch pollen and PFAS to apple causing self-reported visible oropharyngeal angioedema without systemic symptoms such as urticaria or anaphylaxis were included. The exclusion criteria included severe comorbidities (severe asthma and acute illness [eg, illness due to an infection]), use of probiotics, and bariatric surgery because these factors may impose a small additional risk of systemic reactions or may influence the outcome of OIT. The maximum tolerated dose of apple was used as the initial amount for OIT and predetermined with at-home oral provocation tests using Golden Delicious apples, as these contain a high density of *Malus domestica* 1^6^ and are available year round *(*see the Online Repository at www.jaci-global.org). Peeled and grated apple was mixed with 100 mL of yogurt and consumed daily at home in incremental doses according to a standardized schedule (Fig 1 and see the Online Repository). Briefly, the amount of consumed apple was doubled every 3 days for the first 4 steps (1-8 g) and weekly for steps 5 to 8 (16-128 g). After step 8, unpeeled apple was introduced, and yogurt consumption was tapered in 5 days. In cases of angioedema or dyspnea, there were 3 consecutive protocol adaptations, the first being repetition of the previous step. The emergency medication consisted of oral antihistamines, and patients were warned of cofactors such as exercise and use of nonsteroidal anti-inflammatory drugs and alcohol. The main outcomes were the tolerated amount of apple and the time until that amount was reached.

**Fig 1:**
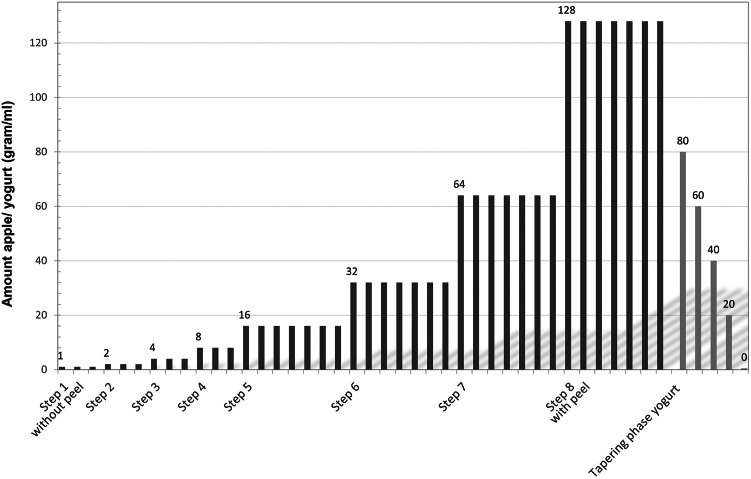
Stepwise protocol of OIT with apple. Each bar represents 1 instance of consumption on 1 day. In phase 1 (*black bars*), the amount of apple in yogurt is gradually increased until 128 g per day. In phase 2 (*gray bars*), the amount of yogurt is tapered from 100 to 0 mL.

Nine patients (8 females with a mean age of 38 years [range 24-54 years] [Table I]) decided to start home OIT. Their median maximum tolerated amount of apple at oral provocation testing was 4 g (range 0-64 g). The OIT protocol was completed by all participants after an average of 39 days (range 19-48 days). The OIT was well tolerated; none of the participants reported dyspnea, systemic symptoms, or angioedema that required treatment. Three participants experienced angioedema, but only 1 of them deemed repetition of 1 protocol step necessary and was subsequently able to continue uneventfully. None of the participants needed to incorporate an additional step or switch apple variety. Eight participants responded to a follow-up questionnaire after 15 months. Of the 8 participants, 6 ate multiple fresh apple cultivars without experiencing any symptoms provided that they kept consuming apples regularly. Two of the 8 were unable to consume 3 apples weekly and lost their initial tolerance. Four participants described an increased tolerance to other fruit of the Rosaceae family, such as peach, pear, cherry, and plum. None of the participants reported symptoms suggestive of eosinophilic esophagitis.^7^

**Table I:** Baseline characteristics of the apple OIT participants

Patient	Sex	Age (y)	Atopic disorders	Airborne allergies	Products causing PFAS	(Food-related) anaphylaxis	MTD	Starting step of OIT
1	F	24	AR, AC, A, AD	Tree pollen, grass pollen, HDM, cat	Apple, pear, kiwi, melon, cherry, plum, tomato, celery, carrot, soy, hazelnut	Yes, to peanut (positive DBPCFC result; Ara h 2 IgE level of 57 kU/L)	2 g	Step 2
2	F	25	AR, A	Tree pollen, grass pollen, HDM, cat	Apple, kiwi, plum, peach, nectarine, almond	No	4 g	Step 3
3	F	43	AR, AC	Tree pollen, grass pollen, HDM, cat, dog	Apple, pear, kiwi, cherry, plum, apricot, walnut	No	0 g	Step 1
4	F	29	AR, AC, AD, SA	Tree pollen, grass pollen, HDM, cat, dog	Apple, kiwi	No	64 g	Step 7
5	F	41	AR, AD	Tree pollen, grass pollen, weed pollen, HDM	Apple, pear, kiwi, cherry, plum, peach, nectarine, carrot, hazelnut, walnut, almond	No	4 g	Step 3
6	F	41	AR, AC, AD	Tree pollen, HDM	Apple, kiwi, cherry, plum, pineapple, tomato, tree nuts	No	8 g	Step 4
7	F	54	AR, AC, AD	Tree pollen, grass pollen, HDM, cat dog, horse, rodents	Apple, pear, kiwi, melon, mango, cherry, plum, pineapple, carrot, hazelnut, walnut	No	0 g	Step 1
8	M	36	AR, AC	Tree pollen	Apple, pear, kiwi, melon, cherry, plum, walnut, pecan	No	0 g	Step 1
9	F	50	AR, AC	Tree pollen, grass pollen, HDM, dog	Apple, strawberry, banana, pear, kiwi, melon, mango, cherry, plum, pineapple, tomato, carrot, soy, wheat, walnut, pecan, almond	Yes, to oats	4 g	Step 3

*A*, Asthma; *AC*, allergic conjunctivitis; *AD*, atopic dermatitis; *AR*, allergic rhinitis; *Ara h*, *Arapis hypogaea*; *DBPCFC*, double-blinded placebo-controlled food challenge; *F*, female, *HDM*, house dust mite; *M*, male; *MTD*, maximum tolerated dose; *SA*, seasonal asthma; *SCIT*, subcutaneous immunotherapy; *SLIT*, sublingual immunotherapy.

To our knowledge, this is the first report of a study in which all participants tolerated a whole apple after OIT. On average, the end point was reached after 39 days, which is considerably sooner than the previously reported time of 140 to 240 days.^4^^,^^5^ This fact may be related to shorter intervals, as we doubled the quantity of apple every 3 to 7 days instead of every 2 to 3 weeks.^5^^,^^6^ Moreover, the patients were allowed to continue OIT if they experienced tingling sensations only; this more liberal approach may have prevented needless delay. In the case of 2 patients, it may be possible that their concurrent subcutaneous immunotherapy for birch pollen facilitated the OIT; however, 1 of the 2 had previously tried a different OIT regimen and aborted it when subcutaneous immunotherapy actually worsened her symptoms. Lastly, we speculate that the addition of yogurt facilitated OIT, presumably by reducing direct contact between the apple and oral mucosa. Alternatively, fermented dairy may positively influence the gut microbiome,^8^ although this is not likely to occur directly or at the study dosages. The *a priori* risk of anaphylaxis in PFAS is very low; we therefore consider it safe to perform OIT apple at home, provided that patients are selected carefully, clear instructions are given, and the dosage of apple is increased gradually. Our results are limited by the small patient numbers, the absence of a control group, the subjectivity of symptoms that is inherent to PFAS, and difficulties in standardizing the allergen dosage. Nonetheless, for all of the participants, there was an obvious increase in the tolerable amount of apple versus at baseline, reflecting a relevant clinical outcome for daily practice. Provided that apple was consumed regularly (we advised ≥3 apples per week), the induced tolerance was sustained and appeared to extend to other apple varieties and stone fruit. These findings suggest that tolerance could be extended to multiple Bet v1 homologues, and it is tempting to speculate that reintroduction of various fruits, vegetables, and tree nuts is feasible. Previous findings by Nothegger et al support this hypothesis.^5^ Because the majority of patients experience PFAS symptoms in response to multiple products and many of them experience considerable dietary restrictions affecting their quality of life,^9^ OIT may have the potential to improve the diet of patients with PFAS beyond apple reintroduction. Increasing variety in the daily diet by reintroducing various fruit and vegetables could facilitate maintaining tolerance to Bet v1 products in the long term.

In our hands, apple OIT appears to be a safe, effective, and rapid method that can be self-conducted at home by patients with PFAS, provided that the patients are carefully selected and instructed and backup support remains available during OIT. These findings require validation in a larger and preferably randomized controlled cohort.

## Disclosure statement

Disclosure of potential conflict of interest: H. N. G. Oude Elberink’s institution has received consultancy fees from ALK-Abelló; in addition, she has received fees for delivering lectures from Chiesi, ALK-Abelló, and Meda; consultancy fees from ALK-Abelló; research support from Novartis, MEDA Pharma, Mead Johnson, ALK-Abelló, Shire, and Chiesi; and payment for developing educational presentations from ALK-Abelló. A. A. J. M. van de Ven has received fees for delivering lectures for Takeda. The rest of the authors declare that they have no relevant conflicts of interest.
