# Supplementary material for: Outcomes of apple oral immunotherapy in pollen food allergy syndrome
Source: J Allergy Clin Immunol Glob. 2024 May 1;3(3):100271. doi: 10.1016/j.jacig.2024.100271 (PMC11131049; doi:10.1016/j.jacig.2024.100271)
Supplement: Supplementary data [file mmc1.docx]

## Online Repository for:

**Outcomes of apple oral immunotherapy in pollen food allergy syndrome**

Brief running title: apple immunotherapy in PFAS

**Authors**

Desie Dijkema MSc^1^, Mirte C. Ruitenbeek BSc^1^, Kirsten Weerstand-Noor BSc^1^, Hanneke N.G. Oude Elberink MD PhD^2^, Annick A.J.M. van de Ven MD PhD^2,3^

**Affiliations**

1. Department of Dietetics, University Medical Centre Groningen, Groningen, the Netherlands

2. Department of Internal Medicine, division of Allergology, University Medical Centre Groningen, Groningen, the Netherlands

3. Department of Rheumatology and Clinical Immunology, University Medical Centre Groningen, Groningen, the Netherlands

**Corresponding author**

Desie Dijkema

Department of Dietetics

Corresponding address AA34

Hanzeplein 1

9713 GZ Groningen

The Netherlands

Phone (secr) +3150-3614178

Fax not available

Email: d.dijkema@umcg.nl

## Information for patients

##

## E1. Protocol of the apple oral provocation test at home

We kindly ask you to perform an oral provocation test (OPT) prior to the initiation of oral immunotherapy (OIT). This means that you will start eating small amounts of apple on day 1, in order to determine your current threshold of apple tolerance.

With this test, your individual starting dose for OIT will be determined. During the oral home introduction test, you will gradually increase your intake of apple until you start experiencing symptoms. **It is pivotal that you perform the OPT exactly according to the instructions below**.

De test will be performed with **peeled** Golden Delicious apple. We chose this apple since it has a high concentration of the allergen to which you react. This means that once you have been able to induce tolerance for this particular apple, you will be able to also tolerate other apple varieties that contain less allergen. Therefore, you must only use Golden Delicious apples during the course of OIT.

- Weigh the proper amount of apple with a digital scale
- Do you experience tingling in the mouth only? Continue the test
- Do you experience angioedema/swollen lips, mouth, throat or difficulty breathing? Stop the test.
- Rinse your mouth with water. It is optional to take antihistamines.
- Note which step you’ve reached and which symptoms you experienced on form E1.2.

Once you’ve finished the OPT, please return this form together with the questionnaire “OIT with apple” by mail or email.

**E1.1 Steps of the apple oral provocation test (OPT)**

**Chew** for a few seconds on the amount of apple and subsequently swallow it.

| **Step** | **Amount of apple** | **Waiting time after eating** |
| --- | --- | --- |
| 1 | 1 gram | Wait for 30 minutes |
| 2 | 2 gram | Wait for 30 minutes |
| 3 | 4 gram | Wait for 30 minutes |
| 4 | 8 gram | Wait for 30 minutes |
| 5 | 16 gram | Wait for 30 minutes |
| 6 | 32 gram | Wait for 30 minutes |
| 7 | 64 gram | Wait for 30 minutes |
| 8 | 128 gram | End of test |

**E1.2 Appel oral provocation test (OPT) score form**

Patient ID:............... date:..................

**Documenting responses**

Document in the “Reaction” column whether there was a reaction and how the reaction manifested itself, for example:

- Itching

- Tingling

- Swelling of the lips, tongue, throat

- tightness

It is important that you describe and record your reactions as completely as possible. Also indicate where the reaction occurred. In the 'reaction duration' box, indicate how long the reaction lasted (in minutes). If you experience swelling symptoms, write down the location of the swelling in the relevant step. **In addition, note that you stopped.**

| **Apple oral provocation test** | | | | |
| --- | --- | --- | --- | --- |
| **Step 1** | **Reaction:** |  |  |  |
|  | 0 none |  |  |  |
|  | 0 itching | 0 tingling | 0 swelling | 0 dyspnea |
|  | *0 lips* | *0 lips* | *0 lips* |  |
|  | *0 tongue* | *0 tongue* | *0 tongue* |  |
|  | *0 palate* | *0 palate* | *0 palate* |  |
|  | *0 throat* | *0 throat* | *0 throat* |  |
|  | *0 other, namely ...* | *0 other, namely ...* | *0 other, namely ...* |  |
|  | **Reaction duration** (minutes): | | | |
| **Step 2** | **Reaction:** |  |  |  |
|  | 0 none |  |  |  |
|  | 0 itching | 0 tingling | 0 swelling | 0 dyspnea |
|  | *0 lips* | *0 lips* | *0 lips* |  |
|  | *0 tongue* | *0 tongue* | *0 tongue* |  |
|  | *0 palate* | *0 palate* | *0 palate* |  |
|  | *0 throat* | *0 throat* | *0 throat* |  |
|  | *0 other, namely ...* | *0 other, namely ...* | *0 other, namely ...* |  |
|  | **Reaction duration** (minutes): | | | |
| **Step 3** | **Reaction:** |  |  |  |
|  | 0 none |  |  |  |
|  | 0 itching | 0 tingling | 0 swelling | 0 dyspnea |
|  | *0 lips* | *0 lips* | *0 lips* |  |
|  | *0 tongue* | *0 tongue* | *0 tongue* |  |
|  | *0 palate* | *0 palate* | *0 palate* |  |
|  | *0 throat* | *0 throat* | *0 throat* |  |
|  | *0 other, namely ...* | *0 other, namely ...* | *0 other, namely ...* |  |
|  | **Reaction duration** (minutes): | | | |
| **Step 4** | **Reaction:** |  |  |  |
|  | 0 none |  |  |  |
|  | 0 itching | 0 tingling | 0 swelling | 0 dyspnea |
|  | *0 lips* | *0 lips* | *0 lips* |  |
|  | *0 tongue* | *0 tongue* | *0 tongue* |  |
|  | *0 palate* | *0 palate* | *0 palate* |  |
|  | *0 throat* | *0 throat* | *0 throat* |  |
|  | *0 other, namely ...* | *0 other, namely ...* | *0 other, namely ...* |  |
|  | **Reaction duration** (minutes): | | | |
| **Step 5** | **Reaction:** |  |  |  |
|  | 0 none |  |  |  |
|  | 0 itching | 0 tingling | 0 swelling | 0 dyspnea |
|  | *0 lips* | *0 lips* | *0 lips* |  |
|  | *0 tongue* | *0 tongue* | *0 tongue* |  |
|  | *0 palate* | *0 palate* | *0 palate* |  |
|  | *0 throat* | *0 throat* | *0 throat* |  |
|  | *0 other, namely ...* | *0 other, namely ...* | *0 other, namely ...* |  |
|  | **Reaction duration** (minutes): | | | |
| **Step 6** | **Reaction:** |  |  |  |
|  | 0 none |  |  |  |
|  | 0 itching | 0 tingling | 0 swelling | 0 dyspnea |
|  | *0 lips* | *0 lips* | *0 lips* |  |
|  | *0 tongue* | *0 tongue* | *0 tongue* |  |
|  | *0 palate* | *0 palate* | *0 palate* |  |
|  | *0 throat* | *0 throat* | *0 throat* |  |
|  | *0 other, namely ...* | *0 other, namely ...* | *0 other, namely ...* |  |
|  | **Reaction duration** (minutes): | | | |
| **Step 7** | **Reaction:** |  |  |  |
|  | 0 none |  |  |  |
|  | 0 itching | 0 tingling | 0 swelling | 0 dyspnea |
|  | *0 lips* | *0 lips* | *0 lips* |  |
|  | *0 tongue* | *0 tongue* | *0 tongue* |  |
|  | *0 palate* | *0 palate* | *0 palate* |  |
|  | *0 throat* | *0 throat* | *0 throat* |  |
|  | *0 other, namely ...* | *0 other, namely ...* | *0 other, namely ...* |  |
|  | **Reaction duration** (minutes): | | | |
| **Step 8** | **Reaction:** |  |  |  |
|  | 0 none |  |  |  |
|  | 0 itching | 0 tingling | 0 swelling | 0 dyspnea |
|  | *0 lips* | *0 lips* | *0 lips* |  |
|  | *0 tongue* | *0 tongue* | *0 tongue* |  |
|  | *0 palate* | *0 palate* | *0 palate* |  |
|  | *0 throat* | *0 throat* | *0 throat* |  |
|  | *0 other, namely ...* | *0 other, namely ...* | *0 other, namely ...* |  |
|  | **Reaction duration** (minutes): | | | |

## E2 Protocol oral immunotherapy with apple

## During this oral immunotherapy, you will be eating Golden Delicious apple in yogurt in small amounts. You will start with the amount of apple that you determined with the home oral provocation test. The amounts will then be step-wise increased. In this way, the chance of reactions is minimized. When a reaction does occur, the reaction will only be mild.

## During the course you will fill in a score form (E2.4), noting the possible reactions. You also note a number between 1-10 (VAS score) to indicate how bad the hay fever symptoms are. Read the additional explanation on the score form. Fill in this figure in the appropriate column on the score form.

## *Why in natural yogurt?*

## Yogurt provides a small layer around the apple, so there is no need for direct contact with the mucosal barrier of the mouth, palate and throat. This makes you less likely to have a reaction, but allows for building tolerance. Also, yogurt is a healthy food.

## *I do not like plain yoghurt, now what?*

## If you do not like plain yoghurt, you can choose to add a little sugar or lemonade to the yoghurt.

## For a maximum of 6 weeks, the amount of food to be introduced is gradually increased. The first 4 steps are increased each time after three days. Steps 5 through 8 are held for 7 days per step, after which increase is made.

## - Wash hands, possibly use gloves if skin complaints arise from touching the apple, or have someone else prepare it for you.

## - Take the amount of apple preferably during day time, so that you can contact your dietician on the same day if you have any complaints or questions.

## - Write down as precisely as possible on the score sheet whether you had any complaints and what kind. You may also want to take a picture of your visible reaction(s). Also write down if you had no complaints.

## *Sending in the score forms*

## It is important that you send in the completed score forms before xx-xx-xxxx. This can be done by mail (xxxxx@umcg.nl) or with the return envelope.

## Have you not yet completed the entire oral immunotherapy? That is not a problem. You can continue the oral immunotherapy as usual. You can send in the completed score forms (until the step you’ve reached at that point), and keep the steps to follow. You can complete these while continuing oral immunotherapy and send them in when you have completed oral immunotherapy.

**Points of attention for apple without peel in yogurt:**

- The test is conducted with a **Golden Delicious** apple, **peeled**.

- Use the same apple for a maximum of 2 days.

- Buy new Golden Delicious apples every week

- Store the purchased apple(s) in the refrigerator

- Always use the same yogurt (brand and type) and also the same amount (100ml)

- Do not let it stand, but eat it immediately

- **Do not eat any brown parts** from the apple.

- Use a digital kitchen scale to weigh the apple. Use the same scale every time.

- Grate the appropriate amount of apple **without the skin** and mix it with the yogurt.

- Add a little sugar if necessary, but do so consistently all days and at every step.

**What should you do if you experience a reaction?**

- Advice for tingling on the lips, in the mouth or throat: continue with the step-by-step plan.

- Advice for tingling with swelling:

1. Rinse your mouth with water or drink 1-2 glasses of water. If necessary, you can take an (extra) tablet of antihistamine. Advice: Take one step back in the scheme and keep it for another 3 days. After that, according to the incremental schedule, try again the step at which the symptoms arose. Example: you experience swelling at step 5. You then go back to the first day of step 4 and continue this step completely, after which you try step 5 again. Contact your dietician if you have any questions.

2. Do you then experience the same reaction again at the same step? You will go back to an amount in between the two steps. Example: You experience swelling at 32 grams of apple. Therefore, you went back to 16 grams and you kept this for 3 days. After three days you tried 32 grams of apple again and again you experience swelling. Advice: You go to the amount between 16 and 32 grams. This is 24 grams. Is this going well? You try after 3 days again 32 grams of apple. Not sure? Consult with the dietician.

3. If you continue to have the reaction at the quantity in question and have done both the above steps, contact your dietician. You will probably be advised to switch to the Elstar apple. This apple contains a lower dose of the allergenic protein that you are reacting to. It could be that you can achieve a higher tolerance with the Elstar apple. Check with the dietitian before you decide to switch to the Elstar.

You will start with the amount to which you had not experienced a reaction of swelling with the Golden Delicious apple.

4. If you experience swelling again with the Elstar at a concerning step, follow up with the adjustment as mentioned above earlier with the Golden Delicious apple (back to advice 1). For questions, contact the dietician.

If you experience reaction and need to go back in the step plan, you can use the 'additional score forms'. How to do this can be found as an explanation of the score forms. If you go through the protocol and have used all of the extra score forms, but you need to make adjustments again at a particular step, you can get extra score forms. To do this, please contact us via email xxxxx@umcg.nl or call 050-xxxxxxx

**E2.1 Incremental schedule for apple in 100 ml yogurt**

Start the incremental schedule with the step that you determined with the home oral provocation test (!). For example: you had no symptoms during the oral home introduction schedule until step 4 (8 grams of apple). You will start at step 4 (week 2, day 3) of this build-up schedule.

| **Step** | **Week** | **Day** | **Amount of apple** |
| --- | --- | --- | --- |
| 1 | 1 | 1 | 1 gram |
|  | 1 | 2 | 1 gram |
|  | 1 | 3 | 1 gram |
| 2 | 1 | 4 | 2 gram |
|  | 1 | 5 | 2 gram |
|  | 1 | 6 | 2 gram |
| 3 | 1 | 7 | 4 gram |
|  | 2 | 1 | 4 gram |
|  | 2 | 2 | 4 gram |
| 4 | 2 | 3 | 8 gram |
|  | 2 | 4 | 8 gram |
|  | 2 | 5 | 8 gram |
| 5 | 2 | 6 | 16 gram |
|  | 2 | 7 | 16 gram |
|  | 3 | 1 | 16 gram |
|  | 3 | 2 | 16 gram |
|  | 3 | 3 | 16 gram |
|  | 3 | 4 | 16 gram |
|  | 3 | 5 | 16 gram |
| 6 | 3 | 6 | 32 gram |
|  | 3 | 7 | 32 gram |
|  | 4 | 1 | 32 gram |
|  | 4 | 2 | 32 gram |
|  | 4 | 3 | 32 gram |
|  | 4 | 4 | 32 gram |
|  | 4 | 5 | 32 gram |
| 7 | 4 | 6 | 64 gram |
|  | 4 | 7 | 64 gram |
|  | 5 | 1 | 64 gram |
|  | 5 | 2 | 64 gram |
|  | 5 | 3 | 64 gram |
|  | 5 | 4 | 64 gram |
|  | 5 | 5 | 64 gram |

**Attention!** The ultimate step is slightly different from the previous steps, as the goal is to be able to eat whole apple. Therefore, at step 8, the apple will be eaten **with peel.**

| **Step** | **Week** | **Day** | **Amount of apple** | **Amount of peel** |
| --- | --- | --- | --- | --- |
| 8 | 5 | 6 | 128 gram | Whole skin |
|  | 5 | 7 | 128 gram | Whole skin |
|  | 6 | 1 | 128 gram | Whole skin |
|  | 6 | 2 | 128 gram | Whole skin |
|  | 6 | 3 | 128 gram | Whole skin |
|  | 6 | 4 | 128 gram | Whole skin |
|  | 6 | 5 | 128 gram | Whole skin |

Once you have completed these steps without any problems, you will now reduce the amount of yogurt. The idea is that you can also eat apple with peel without yogurt.

The yogurt will be reduced in the following manner.

**E2.2 Tapering schedule yoghurt**

| **Tapering**  **week** |  |  | Amount of apple | Amount of yogurt | Amount of skin |
| --- | --- | --- | --- | --- | --- |
| Step 1 | Week 6 | Day 6 | 128 gram | 80 mL | Whole skin |
| Step 2 | Week 6 | Day 7 | 128 gram | 60 mL | Whole skin |
| Step 3 | Week 7 | Day 1 | 128 gram | 40 mL | Whole skin |
| Step 4 | Week 7 | Day 2 | 128 gram | 20 mL | Whole skin |
| Step 5 | Week 7 | Day 3 | 128 gram | No yoghurt | Whole skin |

**What should I do if I experience symptoms while discontinuing yogurt?**

- If you experience symptoms of itching and/or tingling, you can continue with the withdrawal schedule.

- If you experience any swelling, go back to the step with the amount of yogurt you did not experience. Continue this step for 3 days and then follow the phasing out schedule again.

**Maintaining tolerance**

If you have completed the above steps without any problems then continue to eat a 1 whole apple **at least 3 days a week**. This is necessary to maintain tolerance to apple.

**E2.3 Apple oral immunotherapy instructions**

During oral immunotherapy, fill out a score form, documenting the possible reactions, the duration of the reactions, and the VAS Score.

**Documenting reactions**

In the ‘Reaction’ column, note whether there has been a reaction and how the reaction is expressed, for example:

- Itching
- Tingling
- Swelling of the lips, tongue, throat
- Dyspnea

It is important that you write down your reactions as completely as possible. Also indicate **where** the reaction occurred.

In the 'reaction duration' box, indicate how long the reaction lasted (in minutes).

If symptoms of swelling develop and you have to return to a previous step, note on the score sheet at the relevant step that you experienced symptoms of swelling there and that you stopped at this step.

**Visual Analog Scale (VAS):**

In addition to noting your reactions, give a number between 1-10 (VAS score) each day to indicate the extent to which you are currently experiencing hay fever symptoms. You enter this number in the appropriate column on the score sheet.

The VAS score converts a feeling into a number. Try to express the following question as best you can in a number and write it down on the score form:

*To what extent do you currently suffer from hay fever symptoms?*


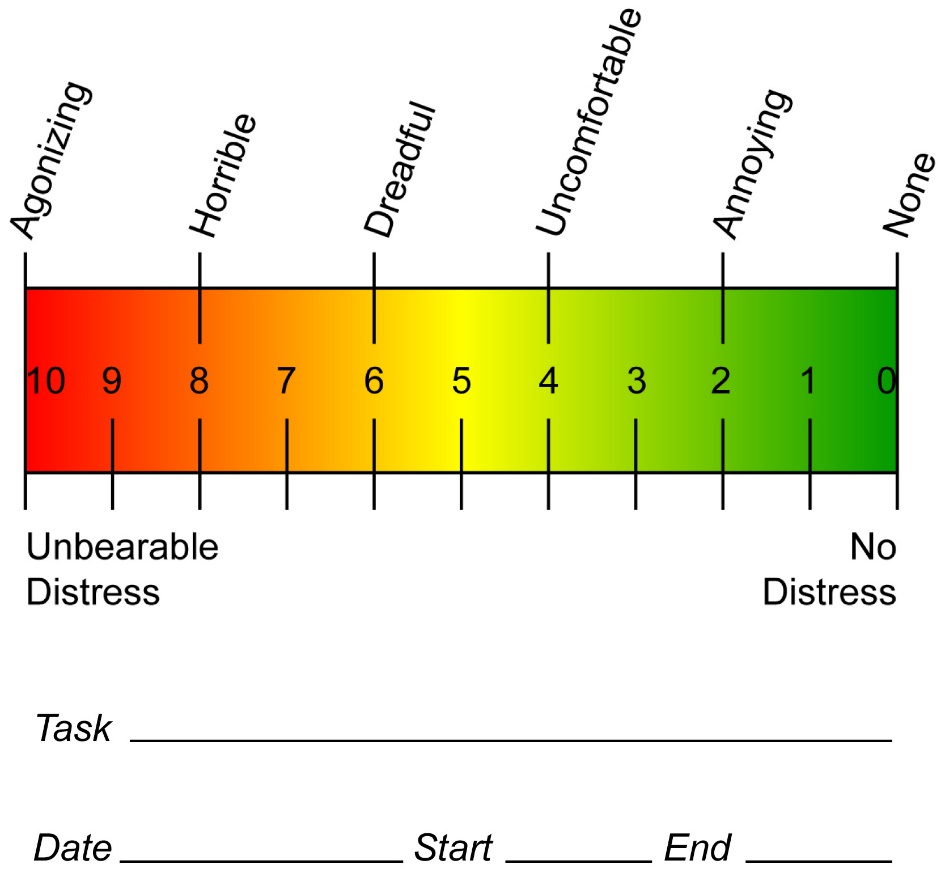


**E2.4 Apple oral immunotherapy score form**

Patient ID ………………. Date:…………………….

| **Step 1:** 1 gram apple 0 Golden Delicious 0 Elstar  *(tick box)* | | | |
| --- | --- | --- | --- |
| **Day 1** | | **Reaction:**  0 none  0 itch 0 tingling 0 swelling 0 dyspnea  *0 lips 0 lips 0 lips*  *0 tongue 0 tongue 0 tongue*  *0 palate 0 palate 0 palate*  *0 throat 0 throat 0 throat*  *0 other, namely…. 0 other, namely…. 0 other, namely….* | |
|  |  | **Reaction duration** (minutes): | |
|  |  | **VAS- score** (1-10): | |
| **Day 2** | | **Reaction:**  0 none  0 itch 0 tingling 0 swelling 0 dyspnea  *0 lips 0 lips 0 lips*  *0 tongue 0 tongue 0 tongue*  *0 palate 0 palate 0 palate*  *0 throat 0 throat 0 throat*  *0 other, namely…. 0 other, namely…. 0 other, namely….* | |
|  |  | **Reaction duration** (minutes): | |
|  |  | **VAS- score** (1-10): | |
| **Day 3** | | **Reaction:**  0 none  0 itch 0 tingling 0 swelling 0 dyspnea  *0 lips 0 lips 0 lips*  *0 tongue 0 tongue 0 tongue*  *0 palate 0 palate 0 palate*  *0 throat 0 throat 0 throat*  *0 other, namely…. 0 other, namely…. 0 other, namely….* | |
|  |  | **Reaction duration** (minutes): | |
|  |  | **VAS- score** (1-10): | |
| **Step 2: 2** gram apple 0 Golden Delicious 0 Elstar  *(tick box)* | | | |
| **Day 1** | | **Reaction:**  0 none  0 itch 0 tingling 0 swelling 0 dyspnea  *0 lips 0 lips 0 lips*  *0 tongue 0 tongue 0 tongue*  *0 palate 0 palate 0 palate*  *0 throat 0 throat 0 throat*  *0 other, namely…. 0 other, namely…. 0 other, namely….* | |
|  |  | **Reaction duration** (minutes): | |
|  |  | **VAS- score** (1-10): | |
| **Day 2** | | **Reaction:**  0 none  0 itch 0 tingling 0 swelling 0 dyspnea  *0 lips 0 lips 0 lips*  *0 tongue 0 tongue 0 tongue*  *0 palate 0 palate 0 palate*  *0 throat 0 throat 0 throat*  *0 other, namely…. 0 other, namely…. 0 other, namely….* | |
|  |  | **Reaction duration** (minutes): | |
|  |  | **VAS- score** (1-10): | |
| **Day 3** | | **Reaction:**  0 none  0 itch 0 tingling 0 swelling 0 dyspnea  *0 lips 0 lips 0 lips*  *0 tongue 0 tongue 0 tongue*  *0 palate 0 palate 0 palate*  *0 throat 0 throat 0 throat*  *0 other, namely…. 0 other, namely…. 0 other, namely….* | |
|  |  | **Reaction duration** (minutes): | |
|  |  | **VAS- score** (1-10): | |
| **Step 3: 4** gram apple 0 Golden Delicious 0 Elstar  *(tick box)* | | | |
| **Day 1** | | **Reaction:**  0 none  0 itch 0 tingling 0 swelling 0 dyspnea  *0 lips 0 lips 0 lips*  *0 tongue 0 tongue 0 tongue*  *0 palate 0 palate 0 palate*  *0 throat 0 throat 0 throat*  *0 other, namely…. 0 other, namely…. 0 other, namely….* | |
|  |  | **Reaction duration** (minutes): | |
|  |  | **VAS- score** (1-10): | |
| **Day 2** | | **Reaction:**  0 none  0 itch 0 tingling 0 swelling 0 dyspnea  *0 lips 0 lips 0 lips*  *0 tongue 0 tongue 0 tongue*  *0 palate 0 palate 0 palate*  *0 throat 0 throat 0 throat*  *0 other, namely…. 0 other, namely…. 0 other, namely….* | |
|  |  | **Reaction duration** (minutes): | |
|  |  | **VAS- score** (1-10): | |
| **Day 3** | | **Reaction:**  0 none  0 itch 0 tingling 0 swelling 0 dyspnea  *0 lips 0 lips 0 lips*  *0 tongue 0 tongue 0 tongue*  *0 palate 0 palate 0 palate*  *0 throat 0 throat 0 throat*  *0 other, namely…. 0 other, namely…. 0 other, namely….* | |
|  |  | **Reaction duration** (minutes): | |
|  |  | **VAS- score** (1-10): | |
| **Step 4: 8** gram apple 0 Golden Delicious 0 Elstar  *(tick box)* | | | |
| **Day 1** | | **Reaction:**  0 none  0 itch 0 tingling 0 swelling 0 dyspnea  *0 lips 0 lips 0 lips*  *0 tongue 0 tongue 0 tongue*  *0 palate 0 palate 0 palate*  *0 throat 0 throat 0 throat*  *0 other, namely…. 0 other, namely…. 0 other, namely….* | |
|  |  | **Reaction duration** (minutes): | |
|  |  | **VAS- score** (1-10): | |
| **Day 2** | | **Reaction:**  0 none  0 itch 0 tingling 0 swelling 0 dyspnea  *0 lips 0 lips 0 lips*  *0 tongue 0 tongue 0 tongue*  *0 palate 0 palate 0 palate*  *0 throat 0 throat 0 throat*  *0 other, namely…. 0 other, namely…. 0 other, namely….* | |
|  |  | **Reaction duration** (minutes): | |
|  |  | **VAS- score** (1-10): | |
| **Day 3** | | **Reaction:**  0 none  0 itch 0 tingling 0 swelling 0 dyspnea  *0 lips 0 lips 0 lips*  *0 tongue 0 tongue 0 tongue*  *0 palate 0 palate 0 palate*  *0 throat 0 throat 0 throat*  *0 other, namely…. 0 other, namely…. 0 other, namely….* | |
|  |  | **Reaction duration** (minutes): | |
|  |  | **VAS- score** (1-10): | |
| **Step 5:** 16 gram apple 0 Golden Delicious 0 Elstar  *(tick box)* | | | |
| **Day 1** | | **Reaction:**  0 none  0 itch 0 tingling 0 swelling 0 dyspnea  *0 lips 0 lips 0 lips*  *0 tongue 0 tongue 0 tongue*  *0 palate 0 palate 0 palate*  *0 throat 0 throat 0 throat*  *0 other, namely…. 0 other, namely…. 0 other, namely….* | |
|  |  | **Reaction duration** (minutes): | |
|  |  | **VAS- score** (1-10): | |
| **Day 2** | | **Reaction:**  0 none  0 itch 0 tingling 0 swelling 0 dyspnea  *0 lips 0 lips 0 lips*  *0 tongue 0 tongue 0 tongue*  *0 palate 0 palate 0 palate*  *0 throat 0 throat 0 throat*  *0 other, namely…. 0 other, namely…. 0 other, namely….* | |
|  |  | **Reaction duration** (minutes): | |
|  |  | **VAS- score** (1-10): | |
| **Day 3** | | **Reaction:**  0 none  0 itch 0 tingling 0 swelling 0 dyspnea  *0 lips 0 lips 0 lips*  *0 tongue 0 tongue 0 tongue*  *0 palate 0 palate 0 palate*  *0 throat 0 throat 0 throat*  *0 other, namely…. 0 other, namely…. 0 other, namely….* | |
|  |  | **Reaction duration** (minutes): | |
|  |  | **VAS- score** (1-10): | |
| **Day 4** | | **Reaction:**  0 none  0 itch 0 tingling 0 swelling 0 dyspnea  *0 lips 0 lips 0 lips*  *0 tongue 0 tongue 0 tongue*  *0 palate 0 palate 0 palate*  *0 throat 0 throat 0 throat*  *0 other, namely…. 0 other, namely…. 0 other, namely….* | |
|  |  | **Reaction duration** (minutes): | |
|  |  | **VAS- score** (1-10): | |
| **Day 5** | | **Reaction:**  0 none  0 itch 0 tingling 0 swelling 0 dyspnea  *0 lips 0 lips 0 lips*  *0 tongue 0 tongue 0 tongue*  *0 palate 0 palate 0 palate*  *0 throat 0 throat 0 throat*  *0 other, namely…. 0 other, namely…. 0 other, namely….* | |
|  |  | **Reaction duration** (minutes): | |
|  |  | **VAS- score** (1-10): | |
| **Day 6** | | **Reaction:**  0 none  0 itch 0 tingling 0 swelling 0 dyspnea  *0 lips 0 lips 0 lips*  *0 tongue 0 tongue 0 tongue*  *0 palate 0 palate 0 palate*  *0 throat 0 throat 0 throat*  *0 other, namely…. 0 other, namely…. 0 other, namely….* | |
|  |  | **Reaction duration** (minutes): | |
|  |  | **VAS- score** (1-10): | |
| **Day 7** | | **Reaction:**  0 none  0 itch 0 tingling 0 swelling 0 dyspnea  *0 lips 0 lips 0 lips*  *0 tongue 0 tongue 0 tongue*  *0 palate 0 palate 0 palate*  *0 throat 0 throat 0 throat*  *0 other, namely…. 0 other, namely…. 0 other, namely….* | |
|  |  | **Reaction duration** (minutes): | |
|  |  | **VAS- score** (1-10): | |
| **Step 6: 32** gram apple 0 Golden Delicious 0 Elstar  *(tick box)* | | | |
| **Day 1** | | **Reaction:**  0 none  0 itch 0 tingling 0 swelling 0 dyspnea  *0 lips 0 lips 0 lips*  *0 tongue 0 tongue 0 tongue*  *0 palate 0 palate 0 palate*  *0 throat 0 throat 0 throat*  *0 other, namely…. 0 other, namely…. 0 other, namely….* | |
|  |  | **Reaction duration** (minutes): | |
|  |  | **VAS- score** (1-10): | |
| **Day 2** | | **Reaction:**  0 none  0 itch 0 tingling 0 swelling 0 dyspnea  *0 lips 0 lips 0 lips*  *0 tongue 0 tongue 0 tongue*  *0 palate 0 palate 0 palate*  *0 throat 0 throat 0 throat*  *0 other, namely…. 0 other, namely…. 0 other, namely….* | |
|  |  | **Reaction duration** (minutes): | |
|  |  | **VAS- score** (1-10): | |
| **Day 3** | | **Reaction:**  0 none  0 itch 0 tingling 0 swelling 0 dyspnea  *0 lips 0 lips 0 lips*  *0 tongue 0 tongue 0 tongue*  *0 palate 0 palate 0 palate*  *0 throat 0 throat 0 throat*  *0 other, namely…. 0 other, namely…. 0 other, namely….* | |
|  |  | **Reaction duration** (minutes): | |
|  |  | **VAS- score** (1-10): | |
| **Day 4** | | **Reaction:**  0 none  0 itch 0 tingling 0 swelling 0 dyspnea  *0 lips 0 lips 0 lips*  *0 tongue 0 tongue 0 tongue*  *0 palate 0 palate 0 palate*  *0 throat 0 throat 0 throat*  *0 other, namely…. 0 other, namely…. 0 other, namely….* | |
|  |  | **Reaction duration** (minutes): | |
|  |  | **VAS- score** (1-10): | |
| **Day 5** | | **Reaction:**  0 none  0 itch 0 tingling 0 swelling 0 dyspnea  *0 lips 0 lips 0 lips*  *0 tongue 0 tongue 0 tongue*  *0 palate 0 palate 0 palate*  *0 throat 0 throat 0 throat*  *0 other, namely…. 0 other, namely…. 0 other, namely….* | |
|  |  | **Reaction duration** (minutes): | |
|  |  | **VAS- score** (1-10): | |
| **Day 6** | | **Reaction:**  0 none  0 itch 0 tingling 0 swelling 0 dyspnea  *0 lips 0 lips 0 lips*  *0 tongue 0 tongue 0 tongue*  *0 palate 0 palate 0 palate*  *0 throat 0 throat 0 throat*  *0 other, namely…. 0 other, namely…. 0 other, namely….* | |
|  |  | **Reaction duration** (minutes): | |
|  |  | **VAS- score** (1-10): | |
| **Day 7** | | **Reaction:**  0 none  0 itch 0 tingling 0 swelling 0 dyspnea  *0 lips 0 lips 0 lips*  *0 tongue 0 tongue 0 tongue*  *0 palate 0 palate 0 palate*  *0 throat 0 throat 0 throat*  *0 other, namely…. 0 other, namely…. 0 other, namely….* | |
|  |  | **Reaction duration** (minutes): | |
|  |  | **VAS- score** (1-10): | |
| **Step 7: 64** gram apple 0 Golden Delicious 0 Elstar  *(tick box)* | | | |
| **Day 1** | | **Reaction:**  0 none  0 itch 0 tingling 0 swelling 0 dyspnea  *0 lips 0 lips 0 lips*  *0 tongue 0 tongue 0 tongue*  *0 palate 0 palate 0 palate*  *0 throat 0 throat 0 throat*  *0 other, namely…. 0 other, namely…. 0 other, namely….* | |
|  |  | **Reaction duration** (minutes): | |
|  |  | **VAS- score** (1-10): | |
| **Day 2** | | **Reaction:**  0 none  0 itch 0 tingling 0 swelling 0 dyspnea  *0 lips 0 lips 0 lips*  *0 tongue 0 tongue 0 tongue*  *0 palate 0 palate 0 palate*  *0 throat 0 throat 0 throat*  *0 other, namely…. 0 other, namely…. 0 other, namely….* | |
|  |  | **Reaction duration** (minutes): | |
|  |  | **VAS- score** (1-10): | |
| **Day 3** | | **Reaction:**  0 none  0 itch 0 tingling 0 swelling 0 dyspnea  *0 lips 0 lips 0 lips*  *0 tongue 0 tongue 0 tongue*  *0 palate 0 palate 0 palate*  *0 throat 0 throat 0 throat*  *0 other, namely…. 0 other, namely…. 0 other, namely….* | |
|  |  | **Reaction duration** (minutes): | |
|  |  | **VAS- score** (1-10): | |
| **Day 4** | | **Reaction:**  0 none  0 itch 0 tingling 0 swelling 0 dyspnea  *0 lips 0 lips 0 lips*  *0 tongue 0 tongue 0 tongue*  *0 palate 0 palate 0 palate*  *0 throat 0 throat 0 throat*  *0 other, namely…. 0 other, namely…. 0 other, namely….* | |
|  |  | **Reaction duration** (minutes): | |
|  |  | **VAS- score** (1-10): | |
| **Day 5** | | **Reaction:**  0 none  0 itch 0 tingling 0 swelling 0 dyspnea  *0 lips 0 lips 0 lips*  *0 tongue 0 tongue 0 tongue*  *0 palate 0 palate 0 palate*  *0 throat 0 throat 0 throat*  *0 other, namely…. 0 other, namely…. 0 other, namely….* | |
|  |  | **Reaction duration** (minutes): | |
|  |  | **VAS- score** (1-10): | |
| **Day 6** | | **Reaction:**  0 none  0 itch 0 tingling 0 swelling 0 dyspnea  *0 lips 0 lips 0 lips*  *0 tongue 0 tongue 0 tongue*  *0 palate 0 palate 0 palate*  *0 throat 0 throat 0 throat*  *0 other, namely…. 0 other, namely…. 0 other, namely….* | |
|  |  | **Reaction duration** (minutes): | |
|  |  | **VAS- score** (1-10): | |
| **Day 7** | | **Reaction:**  0 none  0 itch 0 tingling 0 swelling 0 dyspnea  *0 lips 0 lips 0 lips*  *0 tongue 0 tongue 0 tongue*  *0 palate 0 palate 0 palate*  *0 throat 0 throat 0 throat*  *0 other, namely…. 0 other, namely…. 0 other, namely….* | |
|  |  | **Reaction duration** (minutes): | |
|  |  | **VAS- score** (1-10): | |
| **Step 8: 128** gram apple **with skin** 0 Golden Delicious 0 Elstar  *(tick box)* | | | |
| **Day 1** | | **Reaction:**  0 none  0 itch 0 tingling 0 swelling 0 dyspnea  *0 lips 0 lips 0 lips*  *0 tongue 0 tongue 0 tongue*  *0 palate 0 palate 0 palate*  *0 throat 0 throat 0 throat*  *0 other, namely…. 0 other, namely…. 0 other, namely….* | |
|  |  | **Reaction duration** (minutes): | |
|  |  | **VAS- score** (1-10): | |
| **Day 2** | | **Reaction:**  0 none  0 itch 0 tingling 0 swelling 0 dyspnea  *0 lips 0 lips 0 lips*  *0 tongue 0 tongue 0 tongue*  *0 palate 0 palate 0 palate*  *0 throat 0 throat 0 throat*  *0 other, namely…. 0 other, namely…. 0 other, namely….* | |
|  |  | **Reaction duration** (minutes): | |
|  |  | **VAS- score** (1-10): | |
| **Day 3** | | **Reaction:**  0 none  0 itch 0 tingling 0 swelling 0 dyspnea  *0 lips 0 lips 0 lips*  *0 tongue 0 tongue 0 tongue*  *0 palate 0 palate 0 palate*  *0 throat 0 throat 0 throat*  *0 other, namely…. 0 other, namely…. 0 other, namely….* | |
|  |  | **Reaction duration** (minutes): | |
|  |  | **VAS- score** (1-10): | |
| **Day 4** | | **Reaction:**  0 none  0 itch 0 tingling 0 swelling 0 dyspnea  *0 lips 0 lips 0 lips*  *0 tongue 0 tongue 0 tongue*  *0 palate 0 palate 0 palate*  *0 throat 0 throat 0 throat*  *0 other, namely…. 0 other, namely…. 0 other, namely….* | |
|  |  | **Reaction duration** (minutes): | |
|  |  | **VAS- score** (1-10): | |
| **Day 5** | | **Reaction:**  0 none  0 itch 0 tingling 0 swelling 0 dyspnea  *0 lips 0 lips 0 lips*  *0 tongue 0 tongue 0 tongue*  *0 palate 0 palate 0 palate*  *0 throat 0 throat 0 throat*  *0 other, namely…. 0 other, namely…. 0 other, namely….* | |
|  |  | **Reaction duration** (minutes): | |
|  |  | **VAS- score** (1-10): | |
| **Day 6** | | **Reaction:**  0 none  0 itch 0 tingling 0 swelling 0 dyspnea  *0 lips 0 lips 0 lips*  *0 tongue 0 tongue 0 tongue*  *0 palate 0 palate 0 palate*  *0 throat 0 throat 0 throat*  *0 other, namely…. 0 other, namely…. 0 other, namely….* | |
|  |  | **Reaction duration** (minutes): | |
|  |  | **VAS- score** (1-10): | |
| **Day 7** | | **Reaction:**  0 none  0 itch 0 tingling 0 swelling 0 dyspnea  *0 lips 0 lips 0 lips*  *0 tongue 0 tongue 0 tongue*  *0 palate 0 palate 0 palate*  *0 throat 0 throat 0 throat*  *0 other, namely…. 0 other, namely…. 0 other, namely….* | |
|  |  | **Reaction duration** (minutes): | |
|  |  | **VAS- score** (1-10): | |
| **Tapering schedule yogurt** 0 Golden Delicious 0 Elstar  *(tick box)* | | |  |
| **Day 1**  **80 mL** | **Reaction:**  0 none  0 itch 0 tingling 0 swelling 0 dyspnea  *0 lips 0 lips 0 lips*  *0 tongue 0 tongue 0 tongue*  *0 palate 0 palate 0 palate*  *0 throat 0 throat 0 throat*  *0 other, namely…. 0 other, namely…. 0 other, namely….* | |  |
|  | **Reaction duration** (minutes): | |  |
|  | **VAS- score** (1-10): | |  |
| **Day 2**  **60 mL** | **Reaction:**  0 none  0 itch 0 tingling 0 swelling 0 dyspnea  *0 lips 0 lips 0 lips*  *0 tongue 0 tongue 0 tongue*  *0 palate 0 palate 0 palate*  *0 throat 0 throat 0 throat*  *0 other, namely…. 0 other, namely…. 0 other, namely….* | |  |
|  | **Reaction duration** (minutes): | |  |
|  | **VAS- score** (1-10): | |  |
| **Day 3**  **40 mL** | **Reaction:**  0 none  0 itch 0 tingling 0 swelling 0 dyspnea  *0 lips 0 lips 0 lips*  *0 tongue 0 tongue 0 tongue*  *0 palate 0 palate 0 palate*  *0 throat 0 throat 0 throat*  *0 other, namely…. 0 other, namely…. 0 other, namely….* | |  |
|  | **Reaction duration** (minutes): | |  |
|  | **VAS- score** (1-10): | |  |
| **Day 4**  **20 mL** | **Reaction:**  0 none  0 itch 0 tingling 0 swelling 0 dyspnea  *0 lips 0 lips 0 lips*  *0 tongue 0 tongue 0 tongue*  *0 palate 0 palate 0 palate*  *0 throat 0 throat 0 throat*  *0 other, namely…. 0 other, namely…. 0 other, namely….* | |  |
|  | **Reaction duration** (minutes): | |  |
|  | **VAS- score** (1-10): | |  |
| **Day 5**  **none** | **Reaction:**  0 none  0 itch 0 tingling 0 swelling 0 dyspnea  *0 lips 0 lips 0 lips*  *0 tongue 0 tongue 0 tongue*  *0 palate 0 palate 0 palate*  *0 throat 0 throat 0 throat*  *0 other, namely…. 0 other, namely…. 0 other, namely….* | |  |
|  | **Reaction duration** (minutes): | |  |
|  | **VAS- score** (1-10): | |  |

E3. Evaluation questionnaire following completion of oral immunotherapy

Patient ID:........... date:................

1. Can you describe how you experience the oral immunotherapy with apple?

2. Could you please explain to what extent you felt comfortable following the oral immunotherapy with apple?

3. Did you experience any symptoms during the oral immunotherapy with apple and if so, to what extent did these symptoms affect your daily functioning?

4. In what situations do you notice that you are no longer (or to a lesser extent) allergic to apple?

5. What is your opinion about the current step-by-step plan of oral immunotherapy with apple? (e.g., about the speed of accrual, amounts, etc.)

6. After this experience, would you like to perform oral immunotherapy with other fruits? Why yes/no?

E4. Additional instructions in case of protocol adaptations

## Additional score forms in case of adjustments in the step-by-step plan

## If you need to go back in the step-by-step plan because of swelling symptoms, go back to the previous step (where no swelling occurred) and go through this step again. Then try again the step where swelling symptoms arose.

**E 5.1 Protocol adaptation 1**

| **Step at which swelling began:**……. **I go back to step**:…. …..  **and keep this for … day. Amount (grams):** …………. | | 2^nd^ time |
| --- | --- | --- |
| **Day 1** | **Reaction:**  0 none  0 itch 0 tingling 0 swelling 0 dyspnea  *0 lips 0 lips 0 lips*  *0 tongue 0 tongue 0 tongue*  *0 palate 0 palate 0 palate*  *0 throat 0 throat 0 throat*  *0 other, namely…. 0 other, namely…. 0 other, namely….* | |
|  | **Reaction duration** (minutes): | |
|  | **VAS- score** (1-10): | |
| **Day 2** | **Reaction:**  0 none  0 itch 0 tingling 0 swelling 0 dyspnea  *0 lips 0 lips 0 lips*  *0 tongue 0 tongue 0 tongue*  *0 palate 0 palate 0 palate*  *0 throat 0 throat 0 throat*  *0 other, namely…. 0 other, namely…. 0 other, namely….* | |
|  | **Reaction duration** (minutes): | |
|  | **VAS- score** (1-10): | |
| **Day 3** | **Reaction:**  0 none  0 itch 0 tingling 0 swelling 0 dyspnea  *0 lips 0 lips 0 lips*  *0 tongue 0 tongue 0 tongue*  *0 palate 0 palate 0 palate*  *0 throat 0 throat 0 throat*  *0 other, namely…. 0 other, namely…. 0 other, namely….* | |
|  | **Reaction duration** (minutes): | |
|  | **VAS- score** (1-10): | |
| **(Day 4)** | **Reaction:**  0 none  0 itch 0 tingling 0 swelling 0 dyspnea  *0 lips 0 lips 0 lips*  *0 tongue 0 tongue 0 tongue*  *0 palate 0 palate 0 palate*  *0 throat 0 throat 0 throat*  *0 other, namely…. 0 other, namely…. 0 other, namely….* | |
|  | **Reaction duration** (minutes): | |
|  | **VAS- score** (1-10): | |
| **(Day 5)** | **Reaction:**  0 none  0 itch 0 tingling 0 swelling 0 dyspnea  *0 lips 0 lips 0 lips*  *0 tongue 0 tongue 0 tongue*  *0 palate 0 palate 0 palate*  *0 throat 0 throat 0 throat*  *0 other, namely…. 0 other, namely…. 0 other, namely….* | |
|  | **Reaction duration** (minutes): | |
|  | **VAS- score** (1-10): | |
| **(Day 6)** | **Reaction:**  0 none  0 itch 0 tingling 0 swelling 0 dyspnea  *0 lips 0 lips 0 lips*  *0 tongue 0 tongue 0 tongue*  *0 palate 0 palate 0 palate*  *0 throat 0 throat 0 throat*  *0 other, namely…. 0 other, namely…. 0 other, namely….* | |
|  | **Reaction duration** (minutes): | |
|  | **VAS- score** (1-10): | |
| **(Day 7)** | **Reaction:**  0 none  0 itch 0 tingling 0 swelling 0 dyspnea  *0 lips 0 lips 0 lips*  *0 tongue 0 tongue 0 tongue*  *0 palate 0 palate 0 palate*  *0 throat 0 throat 0 throat*  *0 other, namely…. 0 other, namely…. 0 other, namely….* | |
|  | **Reaction duration** (minutes): | |
|  | **VAS- score** (1-10): | |

Now repeat the step where you experienced the swelling

| **Repeated step where swelling started (nr.):** ……. **Amount (g):**……. | | 2^nd^ time |
| --- | --- | --- |
| **Day 1** | **Reaction:**  0 none  0 itch 0 tingling 0 swelling 0 dyspnea  *0 lips 0 lips 0 lips*  *0 tongue 0 tongue 0 tongue*  *0 palate 0 palate 0 palate*  *0 throat 0 throat 0 throat*  *0 other, namely…. 0 other, namely…. 0 other, namely….* | |
|  | **Reaction duration** (minutes): | |
|  | **VAS- score** (1-10): | |
| **Day 2** | **Reaction:**  0 none  0 itch 0 tingling 0 swelling 0 dyspnea  *0 lips 0 lips 0 lips*  *0 tongue 0 tongue 0 tongue*  *0 palate 0 palate 0 palate*  *0 throat 0 throat 0 throat*  *0 other, namely…. 0 other, namely…. 0 other, namely….* | |
|  | **Reaction duration** (minutes): | |
|  | **VAS- score** (1-10): | |
| **Day 3** | **Reaction:**  0 none  0 itch 0 tingling 0 swelling 0 dyspnea  *0 lips 0 lips 0 lips*  *0 tongue 0 tongue 0 tongue*  *0 palate 0 palate 0 palate*  *0 throat 0 throat 0 throat*  *0 other, namely…. 0 other, namely…. 0 other, namely….* | |
|  | **Reaction duration** (minutes): | |
|  | **VAS- score** (1-10): | |
| **(Day 4)** | **Reaction:**  0 none  0 itch 0 tingling 0 swelling 0 dyspnea  *0 lips 0 lips 0 lips*  *0 tongue 0 tongue 0 tongue*  *0 palate 0 palate 0 palate*  *0 throat 0 throat 0 throat*  *0 other, namely…. 0 other, namely…. 0 other, namely….* | |
|  | **Reaction duration** (minutes): | |
|  | **VAS- score** (1-10): | |
| **(Day 5)** | **Reaction:**  0 none  0 itch 0 tingling 0 swelling 0 dyspnea  *0 lips 0 lips 0 lips*  *0 tongue 0 tongue 0 tongue*  *0 palate 0 palate 0 palate*  *0 throat 0 throat 0 throat*  *0 other, namely…. 0 other, namely…. 0 other, namely….* | |
|  | **Reaction duration** (minutes): | |
|  | **VAS- score** (1-10): | |
| **(Day 6)** | **Reaction:**  0 none  0 itch 0 tingling 0 swelling 0 dyspnea  *0 lips 0 lips 0 lips*  *0 tongue 0 tongue 0 tongue*  *0 palate 0 palate 0 palate*  *0 throat 0 throat 0 throat*  *0 other, namely…. 0 other, namely…. 0 other, namely….* | |
|  | **Reaction duration** (minutes): | |
|  | **VAS- score** (1-10): | |
| **(Day 7)** | **Reaction:**  0 none  0 itch 0 tingling 0 swelling 0 dyspnea  *0 lips 0 lips 0 lips*  *0 tongue 0 tongue 0 tongue*  *0 palate 0 palate 0 palate*  *0 throat 0 throat 0 throat*  *0 other, namely…. 0 other, namely…. 0 other, namely….* | |
|  | **Reaction duration** (minutes): | |
|  | **VAS- score** (1-10): | |

Once you have completed this step without recurrent complaints of swelling, you can continu on the other score form.

You do experience swelling again? Please continu with the score form on the next page.

**E 5.2 Protocol adaptation 2**

*I have completed the previous steps, but I am again experiencing swelling at the particular step.*

You now go to an amount in between both steps. So between the step where you experience no symptoms of swelling and the step where you do experience swelling. Depending on the step you keep this up for 3 or 7 days (see build-up schedule).

| **Step without swelling** (nr.) :……………. **Chosen amount (g):** ………….  **Step with swelling** (nr.): ………….. | |
| --- | --- |
| **Day 1** | **Reaction:**  0 none  0 itch 0 tingling 0 swelling 0 dyspnea  *0 lips 0 lips 0 lips*  *0 tongue 0 tongue 0 tongue*  *0 palate 0 palate 0 palate*  *0 throat 0 throat 0 throat*  *0 other, namely…. 0 other, namely…. 0 other, namely….* |
|  | **Reaction duration** (minutes): |
|  | **VAS- score** (1-10): |
| **Day 2** | **Reaction:**  0 none  0 itch 0 tingling 0 swelling 0 dyspnea  *0 lips 0 lips 0 lips*  *0 tongue 0 tongue 0 tongue*  *0 palate 0 palate 0 palate*  *0 throat 0 throat 0 throat*  *0 other, namely…. 0 other, namely…. 0 other, namely….* |
|  | **Reaction duration** (minutes): |
|  | **VAS- score** (1-10): |
| **Day 3** | **Reaction:**  0 none  0 itch 0 tingling 0 swelling 0 dyspnea  *0 lips 0 lips 0 lips*  *0 tongue 0 tongue 0 tongue*  *0 palate 0 palate 0 palate*  *0 throat 0 throat 0 throat*  *0 other, namely…. 0 other, namely…. 0 other, namely….* |
|  | **Reaction duration** (minutes): |
|  | **VAS- score** (1-10): |
| **(Day 4)** | **Reaction:**  0 none  0 itch 0 tingling 0 swelling 0 dyspnea  *0 lips 0 lips 0 lips*  *0 tongue 0 tongue 0 tongue*  *0 palate 0 palate 0 palate*  *0 throat 0 throat 0 throat*  *0 other, namely…. 0 other, namely…. 0 other, namely….* |
|  | **Reaction duration** (minutes): |
|  | **VAS- score** (1-10): |
| **(Day 5)** | **Reaction:**  0 none  0 itch 0 tingling 0 swelling 0 dyspnea  *0 lips 0 lips 0 lips*  *0 tongue 0 tongue 0 tongue*  *0 palate 0 palate 0 palate*  *0 throat 0 throat 0 throat*  *0 other, namely…. 0 other, namely…. 0 other, namely….* |
|  | **Reaction duration** (minutes): |
|  | **VAS- score** (1-10): |
| **(Day 6)** | **Reaction:**  0 none  0 itch 0 tingling 0 swelling 0 dyspnea  *0 lips 0 lips 0 lips*  *0 tongue 0 tongue 0 tongue*  *0 palate 0 palate 0 palate*  *0 throat 0 throat 0 throat*  *0 other, namely…. 0 other, namely…. 0 other, namely….* |
|  | **Reaction duration** (minutes): |
|  | **VAS- score** (1-10): |
| **(Day 7)** | **Reaction:**  0 none  0 itch 0 tingling 0 swelling 0 dyspnea  *0 lips 0 lips 0 lips*  *0 tongue 0 tongue 0 tongue*  *0 palate 0 palate 0 palate*  *0 throat 0 throat 0 throat*  *0 other, namely…. 0 other, namely…. 0 other, namely….* |
|  | **Reaction duration** (minutes): |
|  | **VAS- score** (1-10): |

Once you are able to complete this step without swelling, you may continu to the step where the swelling first started

| **Repeat step where swelling started (nr.):** ……. **Amount (g):**……. | | 3^rd^ time |
| --- | --- | --- |
| **Day 1** | **Reaction:**  0 none  0 itch 0 tingling 0 swelling 0 dyspnea  *0 lips 0 lips 0 lips*  *0 tongue 0 tongue 0 tongue*  *0 palate 0 palate 0 palate*  *0 throat 0 throat 0 throat*  *0 other, namely…. 0 other, namely…. 0 other, namely….* | |
|  | **Reaction duration** (minutes): | |
|  | **VAS- score** (1-10): | |
| **Day 2** | **Reaction:**  0 none  0 itch 0 tingling 0 swelling 0 dyspnea  *0 lips 0 lips 0 lips*  *0 tongue 0 tongue 0 tongue*  *0 palate 0 palate 0 palate*  *0 throat 0 throat 0 throat*  *0 other, namely…. 0 other, namely…. 0 other, namely….* | |
|  | **Reaction duration** (minutes): | |
|  | **VAS- score** (1-10): | |
| **Day 3** | **Reaction:**  0 none  0 itch 0 tingling 0 swelling 0 dyspnea  *0 lips 0 lips 0 lips*  *0 tongue 0 tongue 0 tongue*  *0 palate 0 palate 0 palate*  *0 throat 0 throat 0 throat*  *0 other, namely…. 0 other, namely…. 0 other, namely….* | |
|  | **Reaction duration** (minutes): | |
|  | **VAS- score** (1-10): | |
| **(Day 4)** | **Reaction:**  0 none  0 itch 0 tingling 0 swelling 0 dyspnea  *0 lips 0 lips 0 lips*  *0 tongue 0 tongue 0 tongue*  *0 palate 0 palate 0 palate*  *0 throat 0 throat 0 throat*  *0 other, namely…. 0 other, namely…. 0 other, namely….* | |
|  | **Reaction duration** (minutes): | |
|  | **VAS- score** (1-10): | |
| **(Day 5)** | **Reaction:**  0 none  0 itch 0 tingling 0 swelling 0 dyspnea  *0 lips 0 lips 0 lips*  *0 tongue 0 tongue 0 tongue*  *0 palate 0 palate 0 palate*  *0 throat 0 throat 0 throat*  *0 other, namely…. 0 other, namely…. 0 other, namely….* | |
|  | **Reaction duration** (minutes): | |
|  | **VAS- score** (1-10): | |
| **(Day 6)** | **Reaction:**  0 none  0 itch 0 tingling 0 swelling 0 dyspnea  *0 lips 0 lips 0 lips*  *0 tongue 0 tongue 0 tongue*  *0 palate 0 palate 0 palate*  *0 throat 0 throat 0 throat*  *0 other, namely…. 0 other, namely…. 0 other, namely….* | |
|  | **Reaction duration** (minutes): | |
|  | **VAS- score** (1-10): | |
| **(Day 7)** | **Reaction:**  0 none  0 itch 0 tingling 0 swelling 0 dyspnea  *0 lips 0 lips 0 lips*  *0 tongue 0 tongue 0 tongue*  *0 palate 0 palate 0 palate*  *0 throat 0 throat 0 throat*  *0 other, namely…. 0 other, namely…. 0 other, namely….* | |
|  | **Reaction duration** (minutes): | |
|  | **VAS- score** (1-10): | |

Once you have completed this step without experiencing swelling, you may continue on the first score form with the consecutive steps.

Do you experience swelling again? Please contact your dietician: she will advice you to continue with a different apple variety, Elstar.

**E 5.3 Protocol adaptation 3**

**Try the step where swelling arose with an Elstar apple**

If you have completed previous steps, but continue to experience swelling at the step in question with the Golden Delicious apple, you will try the step in question with an Elstar apple after consulting your dietitian. You start at the step where **no swelling** occurred and go through it again.

| The amount where no swelling occurred with Golden Delicious: .............  I went through this quantity again and am now trying the next step with Elstar.  **Step where swelling occurred (no.): ......... Number of grams:.......** | | **Elstar** |
| --- | --- | --- |
| **Day 1** | **Reaction:**  0 none  0 itch 0 tingling 0 swelling 0 dyspnea  *0 lips 0 lips 0 lips*  *0 tongue 0 tongue 0 tongue*  *0 palate 0 palate 0 palate*  *0 throat 0 throat 0 throat*  *0 other, namely…. 0 other, namely…. 0 other, namely….* | |
|  | **Reaction duration** (minutes): | |
|  | **VAS- score** (1-10): | |
| **Day 2** | **Reaction:**  0 none  0 itch 0 tingling 0 swelling 0 dyspnea  *0 lips 0 lips 0 lips*  *0 tongue 0 tongue 0 tongue*  *0 palate 0 palate 0 palate*  *0 throat 0 throat 0 throat*  *0 other, namely…. 0 other, namely…. 0 other, namely….* | |
|  | **Reaction duration** (minutes): | |
|  | **VAS- score** (1-10): | |
| **Day 3** | **Reaction:**  0 none  0 itch 0 tingling 0 swelling 0 dyspnea  *0 lips 0 lips 0 lips*  *0 tongue 0 tongue 0 tongue*  *0 palate 0 palate 0 palate*  *0 throat 0 throat 0 throat*  *0 other, namely…. 0 other, namely…. 0 other, namely….* | |
|  | **Reaction duration** (minutes): | |
|  | **VAS- score** (1-10): | |
| **(Day 4)** | **Reaction:**  0 none  0 itch 0 tingling 0 swelling 0 dyspnea  *0 lips 0 lips 0 lips*  *0 tongue 0 tongue 0 tongue*  *0 palate 0 palate 0 palate*  *0 throat 0 throat 0 throat*  *0 other, namely…. 0 other, namely…. 0 other, namely….* | |
|  | **Reaction duration** (minutes): | |
|  | **VAS- score** (1-10): | |
| **(Day 5)** | **Reaction:**  0 none  0 itch 0 tingling 0 swelling 0 dyspnea  *0 lips 0 lips 0 lips*  *0 tongue 0 tongue 0 tongue*  *0 palate 0 palate 0 palate*  *0 throat 0 throat 0 throat*  *0 other, namely…. 0 other, namely…. 0 other, namely….* | |
|  | **Reaction duration** (minutes): | |
|  | **VAS- score** (1-10): | |
| **(Day 6)** | **Reaction:**  0 none  0 itch 0 tingling 0 swelling 0 dyspnea  *0 lips 0 lips 0 lips*  *0 tongue 0 tongue 0 tongue*  *0 palate 0 palate 0 palate*  *0 throat 0 throat 0 throat*  *0 other, namely…. 0 other, namely…. 0 other, namely….* | |
|  | **Reaction duration** (minutes): | |
|  | **VAS- score** (1-10): | |
| **(Day 7)** | **Reaction:**  0 none  0 itch 0 tingling 0 swelling 0 dyspnea  *0 lips 0 lips 0 lips*  *0 tongue 0 tongue 0 tongue*  *0 palate 0 palate 0 palate*  *0 throat 0 throat 0 throat*  *0 other, namely…. 0 other, namely…. 0 other, namely….* | |
|  | **Reaction duration** (minutes): | |
|  | **VAS- score** (1-10): | |

If you have now completed this step without swelling, continue the step-by-step plan with the first score sheet. You will now continue to eat the Elstar apple at the next steps. Make a note on the score form that you continue with Elstar apple. Do you again experience swelling at the relevant step, but now with Elstar? Then continue with the step plan on the next page.

*I have switched to Elstar but I experience swelling again.*

You go back to the step where no complaints of swelling arose and go through this step again. Depending on the step, this is 3 days or 7 days (see incremental schedule).

| **Step at which swelling started:**……. **I go back to step**:…. …..  **Amount (g):** …………. | | **Elstar** |
| --- | --- | --- |
| **Day 1** | **Reaction:**  0 none  0 itch 0 tingling 0 swelling 0 dyspnea  *0 lips 0 lips 0 lips*  *0 tongue 0 tongue 0 tongue*  *0 palate 0 palate 0 palate*  *0 throat 0 throat 0 throat*  *0 other, namely…. 0 other, namely…. 0 other, namely….* | |
|  | **Reaction duration** (minutes): | |
|  | **VAS- score** (1-10): | |
| **Day 2** | **Reaction:**  0 none  0 itch 0 tingling 0 swelling 0 dyspnea  *0 lips 0 lips 0 lips*  *0 tongue 0 tongue 0 tongue*  *0 palate 0 palate 0 palate*  *0 throat 0 throat 0 throat*  *0 other, namely…. 0 other, namely…. 0 other, namely….* | |
|  | **Reaction duration** (minutes): | |
|  | **VAS- score** (1-10): | |
| **Day 3** | **Reaction:**  0 none  0 itch 0 tingling 0 swelling 0 dyspnea  *0 lips 0 lips 0 lips*  *0 tongue 0 tongue 0 tongue*  *0 palate 0 palate 0 palate*  *0 throat 0 throat 0 throat*  *0 other, namely…. 0 other, namely…. 0 other, namely….* | |
|  | **Reaction duration** (minutes): | |
|  | **VAS- score** (1-10): | |
| **(Day 4)** | **Reaction:**  0 none  0 itch 0 tingling 0 swelling 0 dyspnea  *0 lips 0 lips 0 lips*  *0 tongue 0 tongue 0 tongue*  *0 palate 0 palate 0 palate*  *0 throat 0 throat 0 throat*  *0 other, namely…. 0 other, namely…. 0 other, namely….* | |
|  | **Reaction duration** (minutes): | |
|  | **VAS- score** (1-10): | |
| **(Day 5)** | **Reaction:**  0 none  0 itch 0 tingling 0 swelling 0 dyspnea  *0 lips 0 lips 0 lips*  *0 tongue 0 tongue 0 tongue*  *0 palate 0 palate 0 palate*  *0 throat 0 throat 0 throat*  *0 other, namely…. 0 other, namely…. 0 other, namely….* | |
|  | **Reaction duration** (minutes): | |
|  | **VAS- score** (1-10): | |
| **(Day 6)** | **Reaction:**  0 none  0 itch 0 tingling 0 swelling 0 dyspnea  *0 lips 0 lips 0 lips*  *0 tongue 0 tongue 0 tongue*  *0 palate 0 palate 0 palate*  *0 throat 0 throat 0 throat*  *0 other, namely…. 0 other, namely…. 0 other, namely….* | |
|  | **Reaction duration** (minutes): | |
|  | **VAS- score** (1-10): | |
| **(Day 7)** | **Reaction:**  0 none  0 itch 0 tingling 0 swelling 0 dyspnea  *0 lips 0 lips 0 lips*  *0 tongue 0 tongue 0 tongue*  *0 palate 0 palate 0 palate*  *0 throat 0 throat 0 throat*  *0 other, namely…. 0 other, namely…. 0 other, namely….* | |
|  | **Reaction duration** (minutes): | |
|  | **VAS- score** (1-10): | |

Now continu trying the step were the swelling first started.

| **Repeat step where swelling occurred (nr.):** ……. **Amount (g):**……. | | **2^nd^ time Elstar** |
| --- | --- | --- |
| **Day 1** | **Reaction:**  0 none  0 itch 0 tingling 0 swelling 0 dyspnea  *0 lips 0 lips 0 lips*  *0 tongue 0 tongue 0 tongue*  *0 palate 0 palate 0 palate*  *0 throat 0 throat 0 throat*  *0 other, namely…. 0 other, namely…. 0 other, namely….* | |
|  | **Reaction duration** (minutes): | |
|  | **VAS- score** (1-10): | |
| **Day 2** | **Reaction:**  0 none  0 itch 0 tingling 0 swelling 0 dyspnea  *0 lips 0 lips 0 lips*  *0 tongue 0 tongue 0 tongue*  *0 palate 0 palate 0 palate*  *0 throat 0 throat 0 throat*  *0 other, namely…. 0 other, namely…. 0 other, namely….* | |
|  | **Reaction duration** (minutes): | |
|  | **VAS- score** (1-10): | |
| **Day 3** | **Reaction:**  0 none  0 itch 0 tingling 0 swelling 0 dyspnea  *0 lips 0 lips 0 lips*  *0 tongue 0 tongue 0 tongue*  *0 palate 0 palate 0 palate*  *0 throat 0 throat 0 throat*  *0 other, namely…. 0 other, namely…. 0 other, namely….* | |
|  | **Reaction duration** (minutes): | |
|  | **VAS- score** (1-10): | |
| **(Day 4)** | **Reaction:**  0 none  0 itch 0 tingling 0 swelling 0 dyspnea  *0 lips 0 lips 0 lips*  *0 tongue 0 tongue 0 tongue*  *0 palate 0 palate 0 palate*  *0 throat 0 throat 0 throat*  *0 other, namely…. 0 other, namely…. 0 other, namely….* | |
|  | **Reaction duration** (minutes): | |
|  | **VAS- score** (1-10): | |
| **(Day 5)** | **Reaction:**  0 none  0 itch 0 tingling 0 swelling 0 dyspnea  *0 lips 0 lips 0 lips*  *0 tongue 0 tongue 0 tongue*  *0 palate 0 palate 0 palate*  *0 throat 0 throat 0 throat*  *0 other, namely…. 0 other, namely…. 0 other, namely….* | |
|  | **Reaction duration** (minutes): | |
|  | **VAS- score** (1-10): | |
| **(Day 6)** | **Reaction:**  0 none  0 itch 0 tingling 0 swelling 0 dyspnea  *0 lips 0 lips 0 lips*  *0 tongue 0 tongue 0 tongue*  *0 palate 0 palate 0 palate*  *0 throat 0 throat 0 throat*  *0 other, namely…. 0 other, namely…. 0 other, namely….* | |
|  | **Reaction duration** (minutes): | |
|  | **VAS- score** (1-10): | |
| **(Day 7)** | **Reaction:**  0 none  0 itch 0 tingling 0 swelling 0 dyspnea  *0 lips 0 lips 0 lips*  *0 tongue 0 tongue 0 tongue*  *0 palate 0 palate 0 palate*  *0 throat 0 throat 0 throat*  *0 other, namely…. 0 other, namely…. 0 other, namely….* | |
|  | **Reaction duration** (minutes): | |
|  | **VAS- score** (1-10): | |

If you now complete this step without symptoms of swelling, you can continue with the other score form.

Are you experiencing swelling again? Then continue with the score form on the next page.

*I have completed the previous steps, but I am again experiencing swelling at the step in question.*

You now go to an amount in between both steps. So between the step where you experience no symptoms of swelling and the step where you do experience symptoms of swelling. Depending on the step you keep this up for 3 or 7 days.

| **Step without swelling** (nr.) :……………. **Chosen amount (g):** ………….  **Step with swelling** (nr.): ………….. | | | 1^srt^ time | **Elstar** |
| --- | --- | --- | --- | --- |
| **Day 1** | **Reaction:**  0 none  0 itch 0 tingling 0 swelling 0 dyspnea  *0 lips 0 lips 0 lips*  *0 tongue 0 tongue 0 tongue*  *0 palate 0 palate 0 palate*  *0 throat 0 throat 0 throat*  *0 other, namely…. 0 other, namely…. 0 other, namely….* | | | |
|  | **Reaction duration** (minutes): | | | |
|  | **VAS- score** (1-10): | | | |
| **Day 2** | **Reaction:**  0 none  0 itch 0 tingling 0 swelling 0 dyspnea  *0 lips 0 lips 0 lips*  *0 tongue 0 tongue 0 tongue*  *0 palate 0 palate 0 palate*  *0 throat 0 throat 0 throat*  *0 other, namely…. 0 other, namely…. 0 other, namely….* | | | |
|  | **Reaction duration** (minutes): | | | |
|  | **VAS- score** (1-10): | | | |
| **Day 3** | **Reaction:**  0 none  0 itch 0 tingling 0 swelling 0 dyspnea  *0 lips 0 lips 0 lips*  *0 tongue 0 tongue 0 tongue*  *0 palate 0 palate 0 palate*  *0 throat 0 throat 0 throat*  *0 other, namely…. 0 other, namely…. 0 other, namely….* | | | |
|  | **Reaction duration** (minutes): | | | |
|  | **VAS- score** (1-10): | | | |
| **(Day 4)** | | **Reaction:**  0 none  0 itch 0 tingling 0 swelling 0 dyspnea  *0 lips 0 lips 0 lips*  *0 tongue 0 tongue 0 tongue*  *0 palate 0 palate 0 palate*  *0 throat 0 throat 0 throat*  *0 other, namely…. 0 other, namely…. 0 other, namely….* | | |
|  |  | **Reaction duration** (minutes): | | |
|  |  | **VAS- score** (1-10): | | |
| **(Day 5)** | | **Reaction:**  0 none  0 itch 0 tingling 0 swelling 0 dyspnea  *0 lips 0 lips 0 lips*  *0 tongue 0 tongue 0 tongue*  *0 palate 0 palate 0 palate*  *0 throat 0 throat 0 throat*  *0 other, namely…. 0 other, namely…. 0 other, namely….* | | |
|  |  | **Reaction duration** (minutes): | | |
|  |  | **VAS- score** (1-10): | | |
| **(Day 6)** | | **Reaction:**  0 none  0 itch 0 tingling 0 swelling 0 dyspnea  *0 lips 0 lips 0 lips*  *0 tongue 0 tongue 0 tongue*  *0 palate 0 palate 0 palate*  *0 throat 0 throat 0 throat*  *0 other, namely…. 0 other, namely…. 0 other, namely….* | | |
|  |  | **Reaction duration** (minutes): | | |
|  |  | **VAS- score** (1-10): | | |
| **(Day 7)** | | **Reaction:**  0 none  0 itch 0 tingling 0 swelling 0 dyspnea  *0 lips 0 lips 0 lips*  *0 tongue 0 tongue 0 tongue*  *0 palate 0 palate 0 palate*  *0 throat 0 throat 0 throat*  *0 other, namely…. 0 other, namely…. 0 other, namely….* | | |
|  |  | **Reaction duration** (minutes): | | |
|  |  | **VAS- score** (1-10): | | |

If you can complete this quantity without swelling, go back to the step where swelling occurred. See score sheet on next page. Are you experiencing symptoms of swelling again? See page 60.

| **Repeat step where swelling occurred (nr.):** ……. **Amount (g):**……. | | 3^rd^ time **Elstar** |
| --- | --- | --- |
| **Day 1** | **Reaction:**  0 none  0 itch 0 tingling 0 swelling 0 dyspnea  *0 lips 0 lips 0 lips*  *0 tongue 0 tongue 0 tongue*  *0 palate 0 palate 0 palate*  *0 throat 0 throat 0 throat*  *0 other, namely…. 0 other, namely…. 0 other, namely….* | |
|  | **Reaction duration** (minutes): | |
|  | **VAS- score** (1-10): | |
| **Day 2** | **Reaction:**  0 none  0 itch 0 tingling 0 swelling 0 dyspnea  *0 lips 0 lips 0 lips*  *0 tongue 0 tongue 0 tongue*  *0 palate 0 palate 0 palate*  *0 throat 0 throat 0 throat*  *0 other, namely…. 0 other, namely…. 0 other, namely….* | |
|  | **Reaction duration** (minutes): | |
|  | **VAS- score** (1-10): | |
| **Day 3** | **Reaction:**  0 none  0 itch 0 tingling 0 swelling 0 dyspnea  *0 lips 0 lips 0 lips*  *0 tongue 0 tongue 0 tongue*  *0 palate 0 palate 0 palate*  *0 throat 0 throat 0 throat*  *0 other, namely…. 0 other, namely…. 0 other, namely….* | |
|  | **Reaction duration** (minutes): | |
|  | **VAS- score** (1-10): | |
| **(Day 4)** | **Reaction:**  0 none  0 itch 0 tingling 0 swelling 0 dyspnea  *0 lips 0 lips 0 lips*  *0 tongue 0 tongue 0 tongue*  *0 palate 0 palate 0 palate*  *0 throat 0 throat 0 throat*  *0 other, namely…. 0 other, namely…. 0 other, namely….* | |
|  | **Reaction duration** (minutes): | |
|  | **VAS- score** (1-10): | |
| **(Day 5)** | **Reaction:**  0 none  0 itch 0 tingling 0 swelling 0 dyspnea  *0 lips 0 lips 0 lips*  *0 tongue 0 tongue 0 tongue*  *0 palate 0 palate 0 palate*  *0 throat 0 throat 0 throat*  *0 other, namely…. 0 other, namely…. 0 other, namely….* | |
|  | **Reaction duration** (minutes): | |
|  | **VAS- score** (1-10): | |
| **(Day 6)** | **Reaction:**  0 none  0 itch 0 tingling 0 swelling 0 dyspnea  *0 lips 0 lips 0 lips*  *0 tongue 0 tongue 0 tongue*  *0 palate 0 palate 0 palate*  *0 throat 0 throat 0 throat*  *0 other, namely…. 0 other, namely…. 0 other, namely….* | |
|  | **Reaction duration** (minutes): | |
|  | **VAS- score** (1-10): | |
| **(Day 7)** | **Reaction:**  0 none  0 itch 0 tingling 0 swelling 0 dyspnea  *0 lips 0 lips 0 lips*  *0 tongue 0 tongue 0 tongue*  *0 palate 0 palate 0 palate*  *0 throat 0 throat 0 throat*  *0 other, namely…. 0 other, namely…. 0 other, namely….* | |
|  | **Reaction duration** (minutes): | |
|  | **VAS- score** (1-10): | |

If you now complete this step without swelling, you can continue the first score form with the further steps.

*I chose an amount that was between the step without swelling and the step with swelling, only I am experiencing swelling at this self-selected amount. What should I do now?*

You determine again an amount of Elstar apple, which is less than the amount at which swelling occurred, but more than the amount at which you did not experience swelling. The aim here is to build up tolerance

| **Step without swelling** (nr.) :……………. **Chosen amount (g):** ………….  **Step with swelling** (nr.): ………….. | | | 2^nd^ time | **Elstar** |
| --- | --- | --- | --- | --- |
| **Day 1** | **Reaction:**  0 none  0 itch 0 tingling 0 swelling 0 dyspnea  *0 lips 0 lips 0 lips*  *0 tongue 0 tongue 0 tongue*  *0 palate 0 palate 0 palate*  *0 throat 0 throat 0 throat*  *0 other, namely…. 0 other, namely…. 0 other, namely….* | | | |
|  | **Reaction duration** (minutes): | | | |
|  | **VAS- score** (1-10): | | | |
| **Day 2** | **Reaction:**  0 none  0 itch 0 tingling 0 swelling 0 dyspnea  *0 lips 0 lips 0 lips*  *0 tongue 0 tongue 0 tongue*  *0 palate 0 palate 0 palate*  *0 throat 0 throat 0 throat*  *0 other, namely…. 0 other, namely…. 0 other, namely….* | | | |
|  | **Reaction duration** (minutes): | | | |
|  | **VAS- score** (1-10): | | | |
| **Day 3** | **Reaction:**  0 none  0 itch 0 tingling 0 swelling 0 dyspnea  *0 lips 0 lips 0 lips*  *0 tongue 0 tongue 0 tongue*  *0 palate 0 palate 0 palate*  *0 throat 0 throat 0 throat*  *0 other, namely…. 0 other, namely…. 0 other, namely….* | | | |
|  | **Reaction duration** (minutes): | | | |
|  | **VAS- score** (1-10): | | | |
| **(Day 4)** | | **Reaction:**  0 none  0 itch 0 tingling 0 swelling 0 dyspnea  *0 lips 0 lips 0 lips*  *0 tongue 0 tongue 0 tongue*  *0 palate 0 palate 0 palate*  *0 throat 0 throat 0 throat*  *0 other, namely…. 0 other, namely…. 0 other, namely….* | | |
|  |  | **Reaction duration** (minutes): | | |
|  |  | **VAS- score** (1-10): | | |
| **(Day 5)** | | **Reaction:**  0 none  0 itch 0 tingling 0 swelling 0 dyspnea  *0 lips 0 lips 0 lips*  *0 tongue 0 tongue 0 tongue*  *0 palate 0 palate 0 palate*  *0 throat 0 throat 0 throat*  *0 other, namely…. 0 other, namely…. 0 other, namely….* | | |
|  |  | **Reaction duration** (minutes): | | |
|  |  | **VAS- score** (1-10): | | |
| **(Day 6)** | | **Reaction:**  0 none  0 itch 0 tingling 0 swelling 0 dyspnea  *0 lips 0 lips 0 lips*  *0 tongue 0 tongue 0 tongue*  *0 palate 0 palate 0 palate*  *0 throat 0 throat 0 throat*  *0 other, namely…. 0 other, namely…. 0 other, namely….* | | |
|  |  | **Reaction duration** (minutes): | | |
|  |  | **VAS- score** (1-10): | | |
| **(Day 7)** | | **Reaction:**  0 none  0 itch 0 tingling 0 swelling 0 dyspnea  *0 lips 0 lips 0 lips*  *0 tongue 0 tongue 0 tongue*  *0 palate 0 palate 0 palate*  *0 throat 0 throat 0 throat*  *0 other, namely…. 0 other, namely…. 0 other, namely….* | | |
|  |  | **Reaction duration** (minutes): | | |
|  |  | **VAS- score** (1-10): | | |

**E 5.4 Protocol adaptation for tapering yogurt**

If you experience symptoms of swelling while tapering the yogurt, go mayback one step in the schedule. You will go back to the amount of yogurt where you did not experience any symptoms of swelling on apple. This amount is maintained for 3 days. After this, try the step where swelling occurred again.

| **Amount of yogurt where swelling occurred**:....... ... ml  **I am going back to**:.... ..... ml **with**: 0 Golden Delicious 0 Elstar  *(tick box)* | | 2^nd^ time |
| --- | --- | --- |
| **Day 1** | **Reaction:**  0 none  0 itch 0 tingling 0 swelling 0 dyspnea  *0 lips 0 lips 0 lips*  *0 tongue 0 tongue 0 tongue*  *0 palate 0 palate 0 palate*  *0 throat 0 throat 0 throat*  *0 other, namely…. 0 other, namely…. 0 other, namely….* | |
|  | **Reaction duration** (minutes): | |
|  | **VAS- score** (1-10): | |
| **Day 2** | **Reaction:**  0 none  0 itch 0 tingling 0 swelling 0 dyspnea  *0 lips 0 lips 0 lips*  *0 tongue 0 tongue 0 tongue*  *0 palate 0 palate 0 palate*  *0 throat 0 throat 0 throat*  *0 other, namely…. 0 other, namely…. 0 other, namely….* | |
|  | **Reaction duration** (minutes): | |
|  | **VAS- score** (1-10): | |
| **Day 3** | **Reaction:**  0 none  0 itch 0 tingling 0 swelling 0 dyspnea  *0 lips 0 lips 0 lips*  *0 tongue 0 tongue 0 tongue*  *0 palate 0 palate 0 palate*  *0 throat 0 throat 0 throat*  *0 other, namely…. 0 other, namely…. 0 other, namely….* | |
|  | **Reaction duration** (minutes): | |
|  | **VAS- score** (1-10): | |

If you have maintained this step for three days, go back to the step where swelling occurred. See score sheet below.

| **Amount of yogurt at which swelling occurred:** ……. ml  **with:** 0 Golden Delicious 0 Elstar  *(tick box)* | | 2^nd^ time |
| --- | --- | --- |
| **Day:** …….. | **Reaction:**  0 none  0 itch 0 tingling 0 swelling 0 dyspnea  *0 lips 0 lips 0 lips*  *0 tongue 0 tongue 0 tongue*  *0 palate 0 palate 0 palate*  *0 throat 0 throat 0 throat*  *0 other, namely…. 0 other, namely…. 0 other, namely….* | |
|  | **Reaction duration** (minutes): | |
|  | **VAS- score** (1-10): | |

If you now complete this step without swelling, continue with the further steps of the phasing out schedule

E6. Follow-up questionnaire in the maintenance phase after oral immunotherapy

Dear Sir/Madam ...,

Last year you completed the study with OIT apple in yogurt and were able to eat a whole Golden Delicious apple. We would like to know how your symptoms to apple currently are.

Could you please answer the following questions in as much detail as possible and return this by email? (Time: approximately 2-5 minutes). Please feel free to add any other comments if desired.

1. Do you still eat eating apple? If yes,

- Heated apple
- Fresh apple (i.e. not heated, but unprocessed)

*If you no longer eat fresh apple, please proceed to question 8*

2. How many times a week do you eat fresh apple?

3. On average, how many fresh apples do you eat during the week?

4. Which cultivar of fresh apples do you eat (e.g., Elstar, Golden Delicious, Kanzi, Jazz, Granny Smith, etc.)?

5. Do you (ever) have allergic symptoms when eating apples?

- If yes, what symptoms:
- Only itching of the lips/mouth
- Itching of the lips/mouth and throat
- Swelling of the lips
- Swelling of the tongue
- Swelling of the throat
- Coughing
- Other complaints, namely: .....

With which apple varieties (e.g., Elstar, Golden Delicious, Kanzi, Jazz, Granny Smith, ...) and how often do you experience this?

6. Have you also noticed improvement in your allergic symptoms to other fruits since your OIT apple?

- Yes, I have less symptoms with other fruits, namely ............................................................... (please report which fruit and which symptoms you no longer experience now).
- No

7. Do you have any suggestions to improve the treatment of OIT with apple; other than those you indicated earlier?

Additional question in case the answer to question 1 was “no”:

8. Can you tell us why you no longer eat (fresh) apple?
